# Late Gadolinium Enhancemnet imaging of the Left Ventricle in a single breath-hold using multi-slice spiral PSIR imaging at 3T

**DOI:** 10.1186/1532-429X-18-S1-P304

**Published:** 2016-01-27

**Authors:** Iain Pierce, Jennifer Keegan, Ricardo Wage, David Firmin

**Affiliations:** 1 grid.439338.6Cardiovascular BRU, Royal Brompton Hospital, London, United Kingdom; 2 grid.7445.20000000121138111NHLI, Imperial College London, London, United Kingdom

## Background

Late gadolinium enhancement (LGE) imaging is the standard method of assessment of myocardial viability. Conventional Cartesian phase sensitive inversion recovery (PSIR) LGE imaging is usually performed as a stack of 2D slices, with each slice being acquired in a single breath-hold [1]. Repeated breath-holding is time consuming and can be difficult or tiring for patients which can lead to mis-registration between slices. Spiral imaging provides highly efficient k-space coverage, allowing for improvements in acquired resolutions or reductions in acquisition durations.

We have developed a spiral PSIR LGE sequence with non-Cartesian SENSE to acquire a full stack of 2D slices through the left ventricle within a single breath-hold whilst maintaining spatial resolution.

## Methods

A multi-slice spiral PSIR sequence was designed on a Siemens Skyra 3T scanner to provide similar resolution (acquired pixel size 1.4 × 1.4 mm, reconstructed to 0.7 × 0.7 mm) to a conventional Cartesian PSIR sequence. The slice thickness was 8 mm and with a 2 mm slice gap, required 12 slices to cover the LV with acquisition window 132 ms. Fully sampled spiral data required 18 interleaves per slice, each of 9 ms duration. Non-Cartesian SENSE with acceleration rate of 3 was implemented on a Gadgetron [2] and enabled each slice to be reconstructed with 6 interleaves. 1 interleave of all 12 slices of spiral LGE data were acquired on alternate cardiac cycles. Fully sampled coil sensitivity maps were acquired continuously at the beginning of the scan (2.5 s) resulting in a breath-hold duration of approximately 17 cardiac cycles (including dummy cycle). Water-excitation minimised blurring from fat. As each slice is effectively imaged with a different inversion time, proton density data were acquired in the ‘redundant' cardiac cycles to allow PSIR reconstruction.

Spiral PSIR imaging was performed after the completion of the clinical conventional Cartesian PSIR study in 9 patients, with prior consent.

## Results

**Figure 1:**
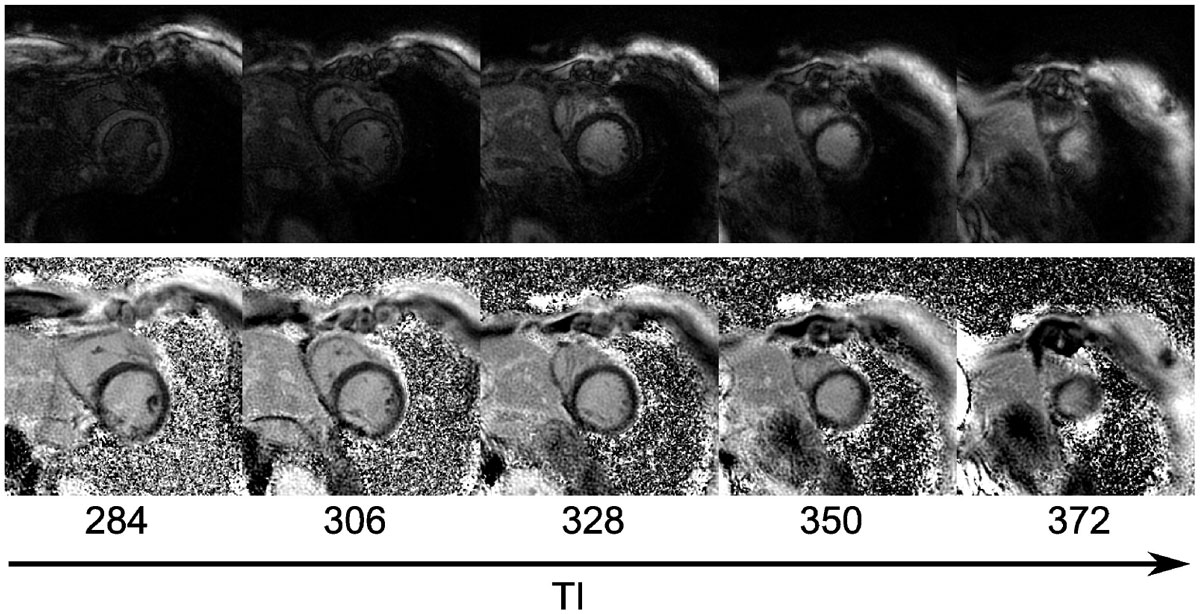
**Magnitude (top) and PSIR (bottom) reconstructions of 5 slices from a multi-slice spiral acquisition**. The inversion time (TI) increases from left to right with PSIR reconstruction necessary to null the myocardium throughout.

**Figure 2:**
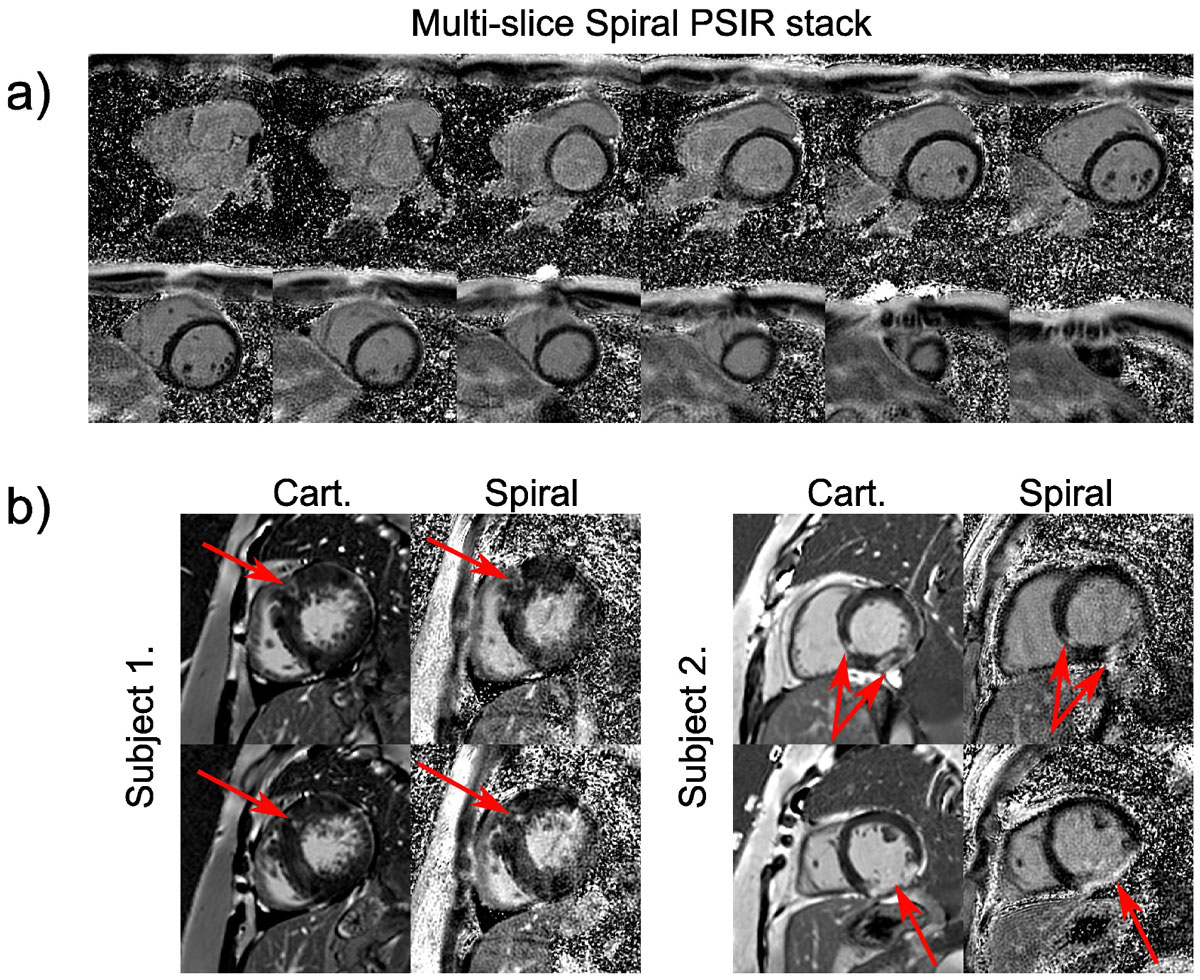
**a) Example multi-slice spiral PSIR LGE images acquired in a single breath-hold from one patient (8 mm slice thickness**. 2 mm gap). b) Example images from 2 patients showing similar regions of enhancement (red arrows) on both Cartesian and multi-slice spiral PSIR images.

Figure 1(a) shows magnitude reconstructions of spiral data from selected slices in a patient showing varying myocardial nulling due to each slice being acquired at a different time after the inversion pulse. The equivalent PSIR reconstructions, 1(b), show good nulling for all slices. Figure 2(a) shows a short axis stack of PSIR images from an example patient. Figure 2(b) shows examples of both Cartesian and spiral PSIR images from 2 patients with regions of enhancement.

## Conclusions

Multi-slice spiral LGE can provide full coverage of the LV in a single breath-hold with the same spatial resolution as a conventional Cartesian multi-breath-hold approach. Each slice is acquired over 11 ms in each cardiac cycle (132 ms for 12 slices), with PSIR reconstruction required to maintain good nulling for all.
